# A novel pan-fungal screening platform for antifungal drug discovery: proof of principle study

**DOI:** 10.1128/aac.01328-24

**Published:** 2025-04-01

**Authors:** Rebecca Inman, Adilia Warris, Elaine Bignell

**Affiliations:** 1 Department of Biosciences, Faculty of Health and Life Sciences, Medical Research Council Center for Medical Mycology at the University of Exeter 98452, Exeter, United Kingdom; University of Iowa, Iowa City, Iowa, USA

**Keywords:** mycology, antifungal susceptibility testing, chemical fragments, antifungal drugs, drug discovery

## Abstract

Broad-spectrum activity is a desirable property of novel antifungal drugs, but relevant *in vitro* testing is complicated by differential nutritional requirements and growth dynamics of fungal pathogens. Many screens for novel drugs are initiated against individual species or genera, with hit compounds later tested for “pan-fungal” activity. Hypothesizing that an optimized pan-fungal methodology would enhance the efficiency of early-stage drug discovery, a standardized assay was developed for a selection of World Health Organization-defined critical and high-priority fungal pathogens. Instead of using the standard susceptibility testing broth RPMI, an enriched media “fungal RPMI” (fRPMI), including multiple additional fungal growth-enhancing nutrients, was utilized. To assess utility for pan-fungal growth assessments, growth in fRPMI was compared to RPMI medium for 12 fungal pathogens. Growth was significantly improved in 7/12 species in fRPMI after 24 and/or 48 hours. For our proof-of-principle study, 500 chemical fragments from the Maybridge Ro3 Fragment library were screened at concentrations of 0.1 or 1 mM against five fungal pathogens: *Aspergillus fumigatus*, *Candida albicans*, *Candida auris*, *Cryptococcus neoformans*, and *Nakaseomyces glabratus*. Assay quality was assessed using *z*-factor analysis, and hits were normalized using a standard *z*-score to identify outliers. All assays achieved a high-quality *z*-factor (≥0.5) with readings at ≤24 hours, allowing the identification of 23 compounds with antifungal activity against at least one fungal species. From these, five compounds were identified as having pan-assay interference or broadly toxic properties. In conclusion, hits identified from pan-fungal phenotypic growth-based assays demonstrate reproducibility in all fungal species tested with carefully optimized conditions and precise timing.

## INTRODUCTION

The necessity and importance of diverse treatment options for fungal infections have become increasingly apparent. The clinical utility of available antifungals is limited by undesirable features such as toxicity, adverse drug-drug interactions, antifungal resistance, and restricted routes of administration (1, 2). These factors, as well as a lack of sensitive diagnostic tools and an increasing number of at-risk patients, contribute to an alarmingly high global fungal disease burden (3).

While no new classes of antifungals have become available for clinical use since 2001, recent years have seen a resurgence in antifungal drug discovery, with three new first-in-class antifungals (fosmanogepix, olorofim, and ibrexafungerp) in the later stages of clinical trials (4). Historically, many antimicrobials have been discovered through purification or modification of natural products from bacteria or fungi: an example being amphotericin B, which was purified from *Streptomyces nodosus* in 1955 (5). On the other hand, the azoles are entirely synthetic, originally based on imidazole chemistry and analog-based drug discovery efforts in the 1960s (6). Although a plethora of useful compounds has been derived from these scaffolds, such as second- and third-generation triazoles, the challenges of novel drug discovery must avoid the recycling of previously discovered active moieties acting upon similar pathways that are vulnerable to common antifungal resistance mechanisms. While azole resistance remains the most common of such mechanisms, species such as *Candida auris* display worrying multi-drug-resistant phenotypes (7). Circumnavigating these resistances through compounds with novel modes of action (MOAs) may be a vital step in improving treatment outcomes.

A more diverse sampling of the chemical space, through small molecules or chemical fragments, can result in the discovery of new first-in-class antifungals. *De novo* or “from the beginning” drug discovery aims to create novel pharmacologically active compounds from the early stages of target identification through to an approved and marketable drug. Discovery approaches can be target based, requiring knowledge of “druggable” targets relevant to disease pathology, or phenotypic, where the focus is on achieving desirable phenotypes such as growth inhibition. Due to its unbiased approach toward what is considered the druggable target space, many first-in-class medicines have been derived from phenotypic screening (8). Fragment-based drug discovery (FBDD) is an example of a powerful *de novo* approach involving libraries of chemical fragments (≤300 Da) with low molecular complexity and weak-binding affinity. Screening concentrations for FBDD start at relatively high concentrations, within the µM–mM range, whereas drug candidates are potent at nM concentrations (9). Fragments are considered as molecular building blocks that can be modified, linked to other fragments, and optimized for increased binding affinity and target specificity. FBDD in the bacterial space utilizes a wide range of techniques for the discovery of novel antimicrobials (10, 11), but FBDD studies screening for antifungal compounds are scarce. Many antifungal screening studies, not limited to FBDD, take the approach of screening against a single species, genera, or virulence phenotypes such as biofilms (12–14). This can be motivated by factors such as the clinical need for antifungal agents against particular species or a perceived lack of practicality in screening large compound libraries against multiple pathogens simultaneously.

Traditional susceptibility testing assay conditions (EUCAST [15, 16] or CLSI [17, 18]) can require incubation times of up to 48 hours for accurate results, representing a limiting factor in high-throughput screening. Furthermore, some species, such as *Candida parapsilosis*, have been identified by the World Health Organization (WHO) as high priorities for research development, yet they demonstrate poor growth under standard EUCAST (15) or CLSI (17) conditions. Thus, we set out to develop a standardized pan-fungal screening platform for antifungal drug discovery that is more amenable to high-throughput screening. In this proof of principle study, a selection of WHO-defined critical and high-priority fungal pathogens (19) was chosen for pilot screens against a library of 500 chemical fragments: *Aspergillus fumigatus*, *Candida albicans*, *C. auris*, *Nakaseomyces glabratus* (formerly *Candida glabrata*), and *Cryptococcus neoformans*.

## RESULTS

### Assay optimization and susceptibility testing

**Fig 1:**
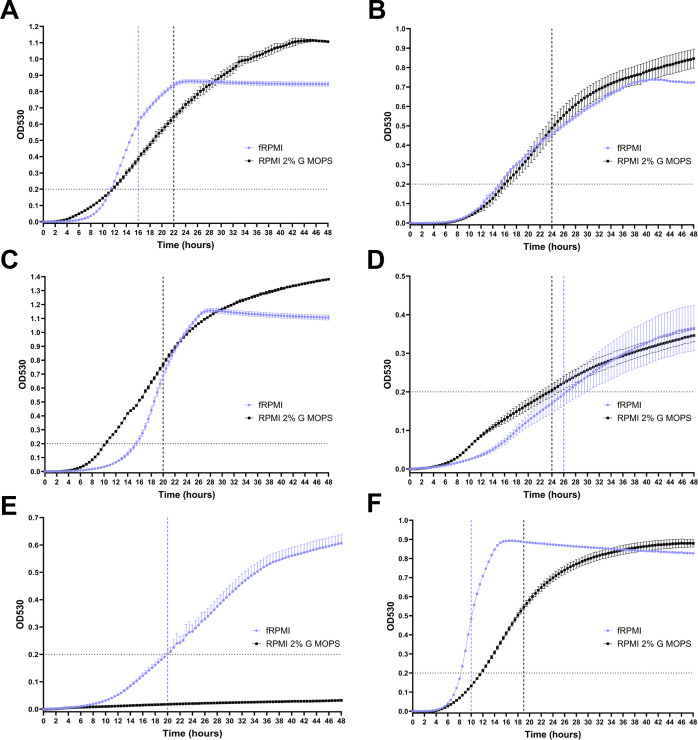
Forty-eighty-hour time-course kinetic growth curves and media comparison for yeast species. Horizontal dotted lines on the y-axis represent an OD threshold of 0.2. Black lines represent RPMI 2% G-MOPS, and purple lines represent fRPMI. Vertical dotted lines on the x-axis identify the approximate optimal measurement times (*T*_*a*_) for each media. A single vertical black line represents an overlap of *T*_*a*_ in both media. Where there is no *T*_*a*_ for the media, the line is omitted. Individual species are represented in A–F as 1A *C. albicans* SC5314, 1B *C. auris* 470036, 1C *N. glabratus* BG2, 1D *C. neoformans* H99E, 1E *C. parapsilosis* NCPF 3207, and 1F *Candida tropicalis* NCPF 3112. Data points represent the mean, and error bars represent ±SD of 3–4 biological replicates.

To achieve a high-quality assay for screening chemical compounds, it was necessary to optimize growth conditions for multiple fungal species. An optimized pan-fungal assay would incorporate three essential elements: simplicity, a medium supporting active pan-fungal growth, and a time scale amenable to high-throughput screening. An optical density (OD) threshold of ≥0.2 above the background levels (recommended by EUCAST [15] for species with poor growth) was set to indicate sufficient growth for yeasts and molds, which had particular relevance to slow-growing species such as *C. neoformans*. An additional criterion for molds was avoiding growth at the liquid-surface interface, which results in large variations in OD measurements. Thus, we selected 6 yeast and six mold species (Table 1) to assess and compare growth conditions in fungal RPMI (fRPMI (20, 21) and RPMI 2% G-3-(N-morpholino) propanesulfonic acid (MOPS) over 48 hours. Key variables for optimization were temperature, time, and conditions of incubation such as agitation and the use of CO_2_. Optimal time-points for growth measurements (hereafter designated as *T*_*a*_) for yeasts were determined using kinetic growth curves (Fig. 1). For yeasts, *T*_*a*_ was represented by the mid-to-late stages of the log growth phase with OD_530_ measurements of 0.4–0.6, or ≥0.2 for slow/poorly growing species. For molds, *T*_*a*_ would be a time-point before OD_600_ readings caused significant SD between replicates or when the OD_600_ ≥ 0.2 threshold was reached.

**TABLE 1:** List of organisms used for kinetic growth curves/pan-fungal screening optimization

Organism	Strain	Source	Reference
*A. fumigatus*	A1160^+^ (Δ*Ku80 pyrG^+^*)	Laboratory derivative (A1163)	(22)
*Aspergillus flavus*	CBS 128202	Environmental (food)	(23)
*Aspergillus niger*	Unknown	Unknown	
*Aspergillus terreus*	Unknown	Environmental	
*Lichtheimia corymbifera*	NCPF 2710	Clinical (orbital)	
*Rhizopus microsporus*	FP469-12	Clinical	(24)
*C. albicans*	SC5314	Clinical (candidiasis)	(25)
*C. auris*	470036 (Clade I)	Clinical (blood)	(26)
*N. glabratus* (formerly *Candida glabrata*)	BG2	Laboratory (parental strain B)	(27)
*C. parapsilosis*	NCPF 3207	Clinical (blood)	
*Candida tropicalis*	NCPF 3112	Clinical (lung)	
*C. neoformans*	H99E	Laboratory passage (H99)	(28)

*T*_*a*_ for yeasts was generally between 10 and 26 hours in fRPMI and 18–24 hours in RPMI 2% G-MOPS (Fig. 1A through F). Growth curves in fRPMI revealed a rapidly accelerated log phase compared to RPMI 2% G-MOPS, reaching a stationary phase plateau in as little as 15 hours for *C. tropicalis*. Compared to the other yeast species, *C. tropicalis* exhibited an exceptionally rapid growth rate in both media: with *T*_*a*_ = 9–10 hours in fRPMI and 18–20 hours in RPMI 2% G-MOPS (Fig. 1F). *C. neoformans* did not meet the ≥0.2 threshold until 24–26 hours (Fig. 1D), but growth conditions, such as incubation at 30°C and/or the addition of 5% CO_2_, did not significantly improve the growth of this organism (data not shown). *C. parapsilosis* was unable to grow in standard RPMI 2% G-MOPS media after 48 hours incubation but reached an acceptable level of growth (OD_530_ ≥ 0.2) after 20 hours in fRPMI (Fig. 1E). End-point growth comparisons revealed that *C. albicans*, *C. tropicalis*, and *C. parapsilosis* displayed significantly improved growth in fRPMI after 24 hours compared to RPMI 2% G-MOPS (Fig. 3A). For *C. auris* and *C. neoformans*, growth levels showed no significant difference at either 24- or 48-hour OD_530_ end-point readings. Thus, fRPMI could be confidently selected as a suitable media for accelerated fungal growth in higher throughput screening.

**Fig 2:**
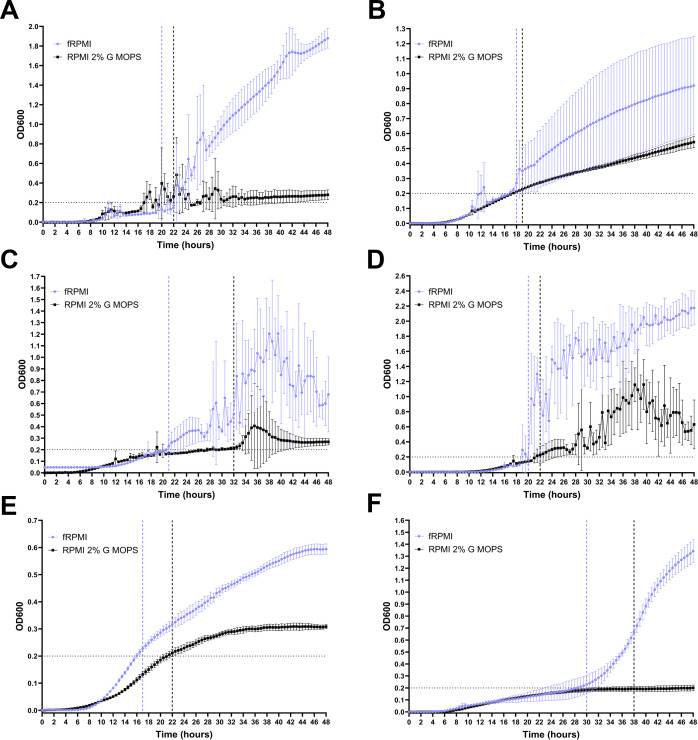
Forty-eighty-hour time-course kinetic growth curves and media comparison for mold species. Horizontal dotted lines on the y-axis represent an OD threshold of 0.2. Black lines represent RPMI 2% G-MOPS, and purple lines represent fRPMI. Vertical dotted lines on the x-axis represent the approximate optimal measurement times (*T*_*a*_) for each media. Individual species are represented in A–F as 2A *A*. *fumigatus* A1160^+^, 2B *Aspergillus flavus* CBS 128202, 2C *Aspergillus niger* 1D *Aspergillus terreus*, 1E *L. corymbifera* NCPF 2710, and 1F *Rhizopus microsporus* FP469-12 NCPF 3207. Data points represent the mean, and error bars represent ±SD of 3–4 biological replicates.

**Fig 3:**
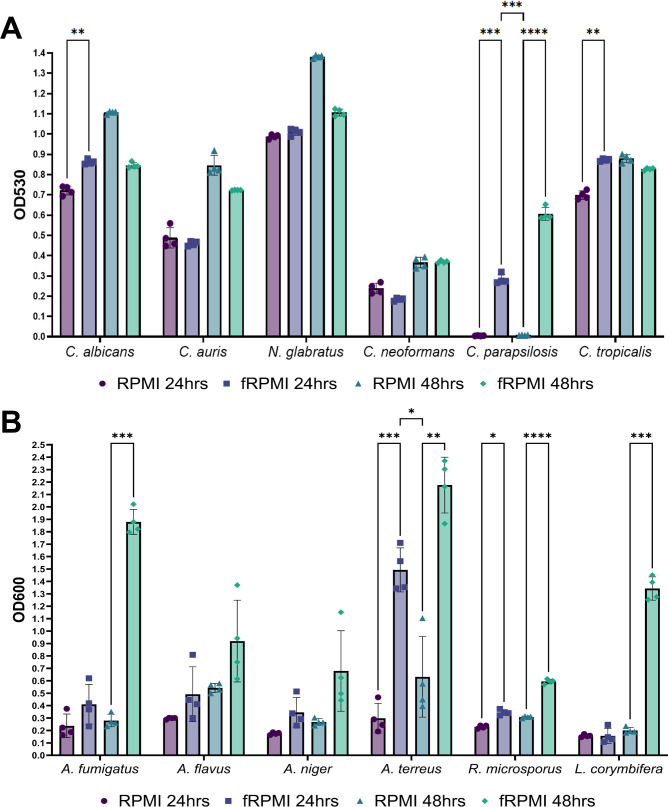
Endpoint growth comparisons for six yeast species (A) and six mold species (B) in fRPMI and RPMI 2% G-MOPS at 24 and 48 hours. Colored bars and symbols represent the mean and data points from individual replicates for each species and media conditions, respectively. Error bars represent ±SD of 3–4 biological replicates. Statistical significance was determined by two-way ANOVA with post-hoc Tukey’s test ****P ≤ 0.0001, ***P ≤ 0.001, **P ≤ 0.01, and *P ≤ 0.05

In a similar manner to yeasts, the kinetic growth curves for molds were used to estimate optimal time-points for OD_600_ measurements (Fig. 2A through F). For *A. fumigatus*, *A. flavus*, *A. niger*, *A. terreus*, *and R. microsporus T*_*a*_ ranged from 17 to 21 hours for fRPMI and 19–32 hours for RPMI 2% G-MOPS (Fig. 2A through E). *Lichtheimia corymbifera* was the slowest growing mold, but sufficient growth could be achieved in ≤30 hours for fRPMI and ≥38 hours for RPMI 2% G-MOPS (Fig. 2F). Overall, growth was poorer in RPMI 2% G-MOPS compared to fRPMI, yet it is still produced high SDs in *A. fumigatus*, *A. niger*, and *A. terreus* (Fig. 2A, C and D). End-point OD_600_ measurements for *A. fumigatus*, *A. terreus*, *R. microsporus*, and *L. corymbifera* were significantly improved in fRPMI compared to RPMI 2% G-MOPS after 24 and/or 48 hours (Fig. 3B). Thus, fRPMI was deemed a suitable media for growth of molds in higher throughput screening.

Optimized assays for *A. fumigatus*, *C. albicans*, *C. auris*, *N. glabratus*, and *C. neoformans* were selected for higher throughput screening. Susceptibility testing against amphotericin B or voriconazole for each of the five fungal organisms provided a positive control (i.e., no growth signal) for the chemical compound screening assays. MIC_90_ and MIC_80_ values for RPMI 2% G-MOPS and fRPMI are provided in Table S1. Generally, MIC_90_/MIC_80_ values between RPMI 2% G-MOPS did not differ from fRPMI by more than onefold dilution. Since an MIC_90_ or complete growth inhibition could not be achieved with voriconazole in the yeast species, amphotericin B was selected as the antifungal control.

### *Z*-factors indicate high assay quality

To determine if the hits could be identified with a high degree of confidence, it was essential to assess the quality of the assay. Hence, a simple statistical parameter known as a *z*-factor was calculated for each organism (29). *Z*-factors indicated an excellent quality assay (≥0.5) for all organisms: 0.58 (0.55–0.61) for *A. fumigatus*, 0.84 (0.83–0.85) for *C. albicans*, 0.85 (0.82–0.87) for *C. auris*, 0.86 (0.84–0.89) for *N. glabratus*, and 0.63 (0.6–0.66) for *C. neoformans*. Details of plate-to-plate variation in Z-factors are shown in Table S2. *A. fumigatus and C. neoformans* assays scored the lowest due to poorer growth or greater OD variability. Despite careful optimization, the non-uniform growth of *A. fumigatus* would occasionally cause higher SDs in the data set, which is one of the major technical difficulties in growth-based assays for filamentous fungi.

### *Z*-score analysis identifies compound hits

**Fig 4:**
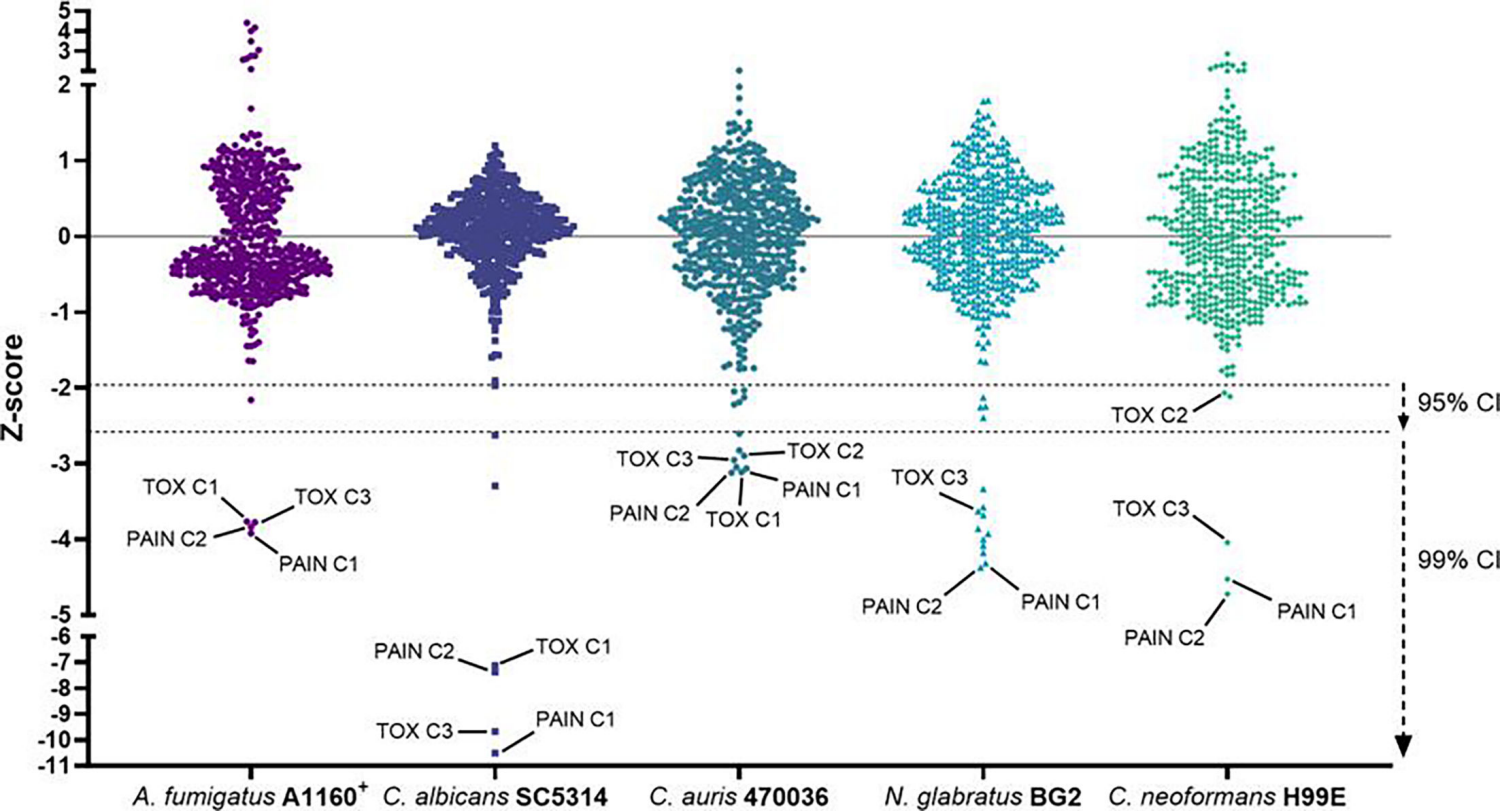
*Z*-score plots for full chemical fragment library screens. Data points represent the *z*-scores calculated from three biological replicates. Pan-assay interference and toxic compounds are indicated as PAIN C1-2 and TOX C1-3, respectively. Dotted lines at −1.96 and −2.58 indicate the threshold for outliers indicating growth reduction with 95% and 99% confidence, respectively.

A *z*-score threshold of ≤−1.96 was selected to indicate growth reduction with 95% confidence, assuming normal distribution on a standard bell curve. A score of ≤−2.58 indicates 99% confidence, but extremely low *z*-scores across multiple organisms were usually indicative of either broadly toxic or pan-assay interference compounds (PAINs; Fig. 4; Table S3). As well as screening at 0.1 mM, *C. neoformans* was screened at a higher concentration of 1 mM. This produced an overwhelmingly large number of hits (133/500) inhibiting growth by ≥50% (Fig. S1), causing the *z*-score analysis to be incomparable to the 0.1 mM screen since the heavy bias in the data set made it impossible to distinguish outliers. For this reason, we did not screen any other organism using this higher concentration as it would most likely result in similarly skewed data.

Chemical names and structures of compound hits were identified using Simplified Molecular Input Line Entry System (SMILES) and are shown in Table 2. The *N. glabratus* screen yielded the largest number of significant hits, with 12/18 identified compounds exhibiting the lowest *z*-scores in *N. glabratus* (−4.2 to −2.2) compared to the other four organisms. Percent inhibition for these compounds ranges from 61.8% to 95% compared to the untreated (dimethyl sulfoxide [DMSO]) control. Conversely, the lowest *z*-score (−2.6) for *C. albicans* corresponded to only 23.9% inhibition, emphasizing the importance of considering both analyses when identifying hits. The full percent inhibition plots for each species at 0.1 mM are shown in Fig. S2. Assessing both percent inhibition and *z*-scores, it appears that some compounds have multi-species activity. Compounds 3 and 4 in particular seem to have activity across all species tested, although exceptionally potent compounds for *C. neoformans* and *C. albicans* remained elusive.

**TABLE 2:** Compound hits with corresponding *z*-scores, percent inhibition compared to untreated control, chemical names, and structures^*a*^

Organism	*Z*-score	% inhibition vs untreated control	Chemical name	Chemical structure
***A. fumigatus***	**−2.2**	**64.8**	**Compound 1** 1-{2-(4-[Trifluoromethoxy]phenyl)−1,3-thiazol-4-yl}ethanone	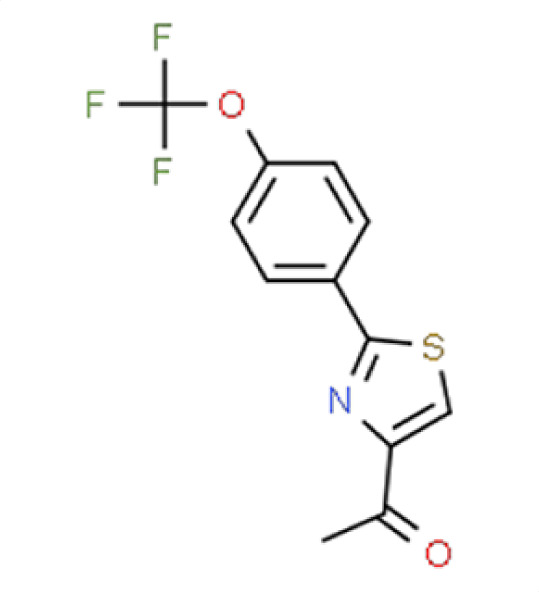
*C. albicans*	−0.5	3.9		
*C. auris*	−0.7	36.4		
*N. glabratus*	−1	21.5		
*C. neoformans*	−1.8	42.7		
*A. fumigatus*	−1.6	53.2	**Compound 2** 2-(4-Methoxyphenyl)−2,3-dihydro-1,3-benzoxazole	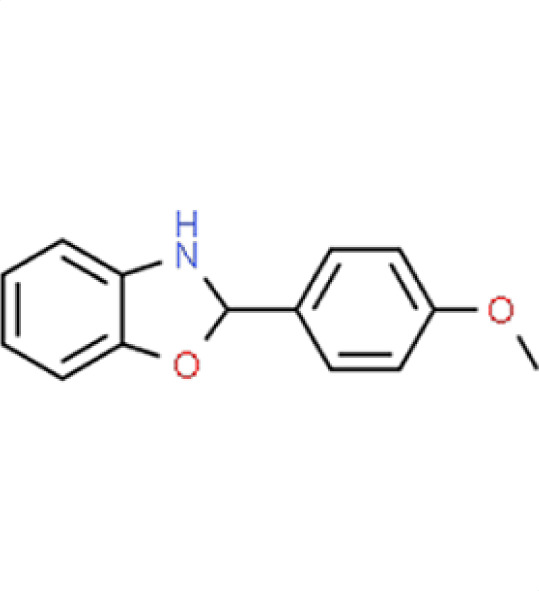
*C. albicans*	0.4	0.2		
*C. auris*	−0.8	32.2		
***N. glabratus***	**−3.6**	**84.2**		
*C. neoformans*	0.5	1.4		
*A. fumigatus*	−0.3	29.9	**Compound 3** 1-(2-[3-Chlorophenyl]−1,3-thiazol-4-yl)-N-methylmethanamine	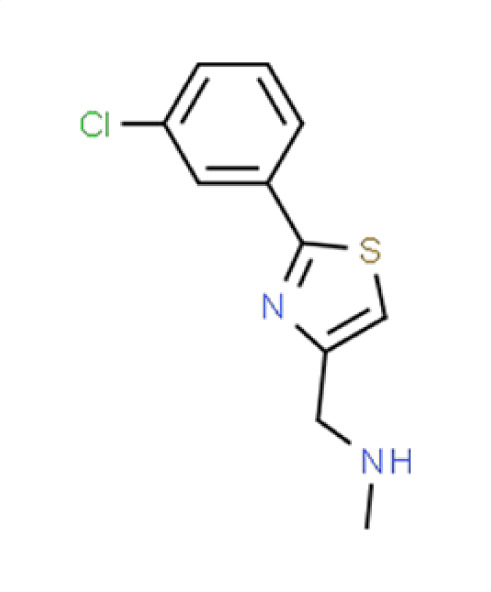
*C. albicans*	−2.6	23.9		
*C. auris*	−2.6	86.1		
***N. glabratus***	**−4.1**	**92.3**		
*C. neoformans*	−0.5	19.5		
*A. fumigatus*	−1.1	46.8	**Compound 4** 1-(3-Chloro-5-[trifluoromethyl]pyridin-2-yl)−1,4-diazepane	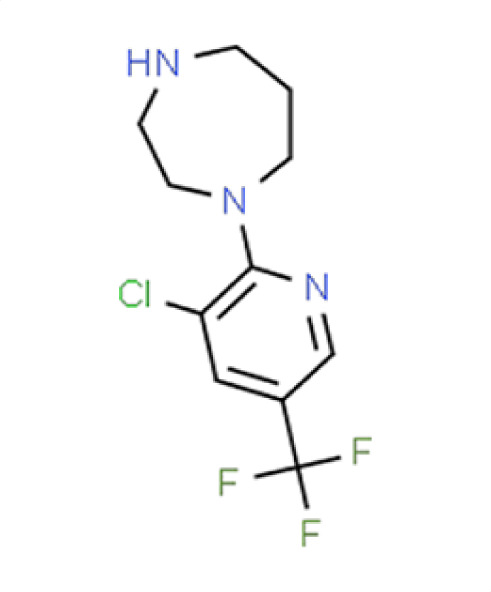
*C. albicans*	−3.3	32.4		
*C. auris*	−2.1	71.6		
***N. glabratus***	**−4.2**	**95**		
*C. neoformans*	−0.6	23		
*A. fumigatus*	0.4	37.2	**Compound 5** 4'-Hydroxydeoxybenzoin	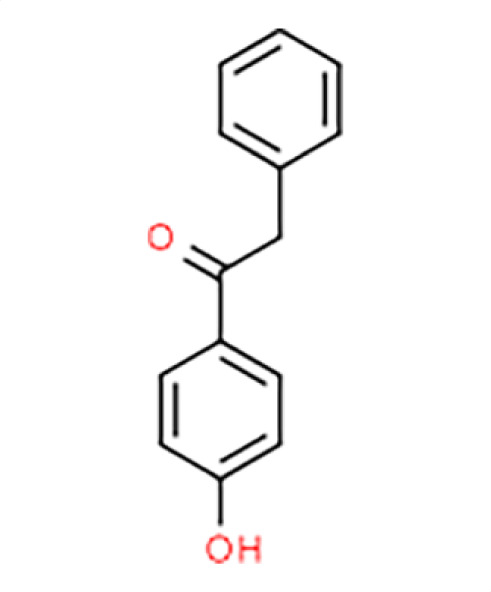
*C. albicans*	−0.4	5.7		
*C. auris*	−0.1	6.4		
*N. glabratus*	−1	50		
***C. neoformans***	**−2.1**	**46.6**		
*A. fumigatus*	−0.9	38.5	**Compound 6** 2-([2-Chloro-6-fluorobenzyl]sulfanyl)−4,5-dihydro-1H-imidazol-3-ium bromide	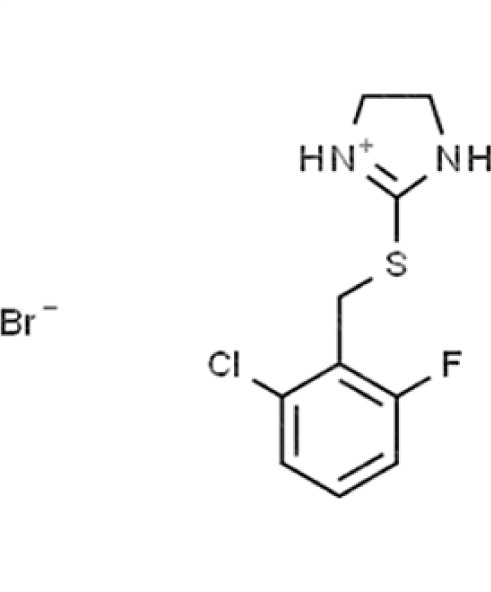
*C. albicans*	−0.9	10.7		
*C. auris*	−2.8	91.3		
***N. glabratus***	**−3.9**	**89.7**		
*C. neoformans*	0.2	8.3		
*A. fumigatus*	0.3	14.5	**Compound 7** 1-Butyl-3-(3-methoxyphenyl)thiourea	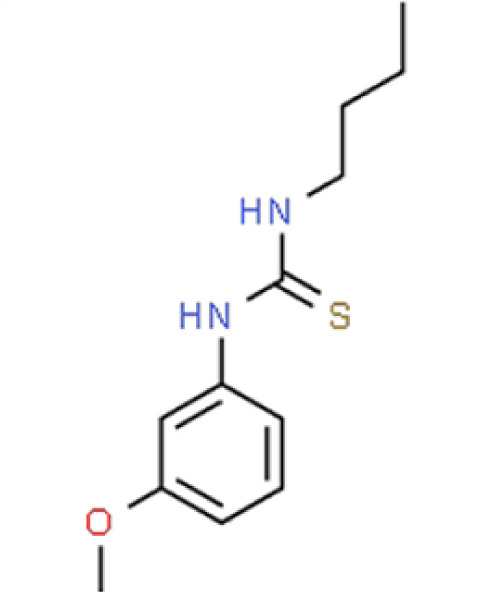
*C. albicans*	−1.6	13.8		
*C. auris*	−0.4	28.4		
***N. glabratus***	**−3.3**	**75**		
*C. neoformans*	−0.1	18		
*A. fumigatus*	−0.8	35.9	**Compound 8** N-(2-Thienylmethyl) hydrazinecarbothioamide	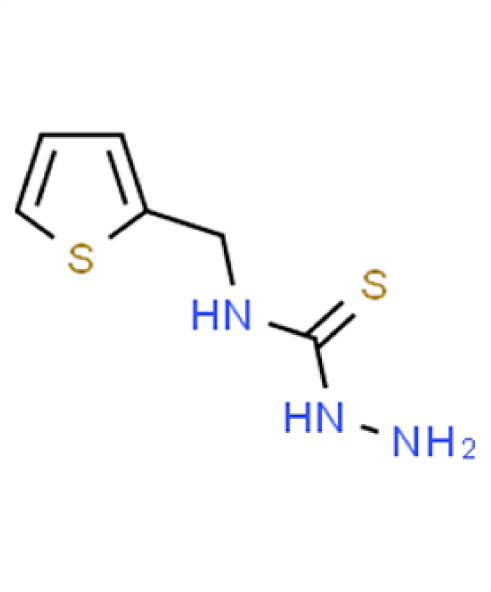
*C. albicans*	−0.9	15.9		
*C. auris*	−3	97.5		
***N. glabratus***	**−3.9**	**89.6**		
*C. neoformans*	−0.7	22.8		
*A. fumigatus*	−0.7	36	**Compound 9** N-methyl-N-(4-thien-2-ylbenzyl)amine	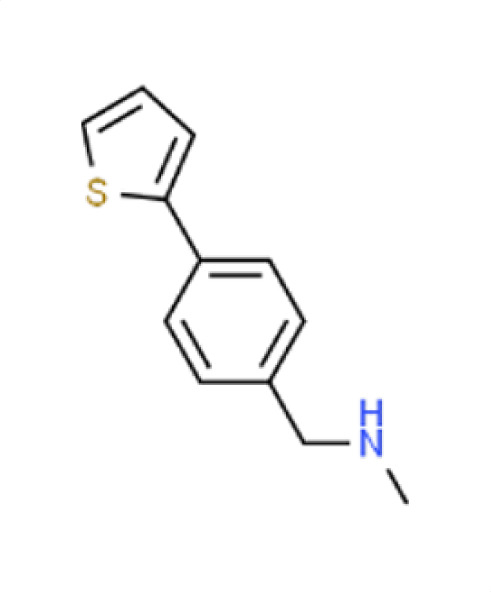
*C. albicans*	−1	11.6		
*C. auris*	−1.3	44.1		
***N. glabratus***	**−4**	**93.2**		
*C. neoformans*	0.8	−11.6		
*A. fumigatus*	2.6	−5.5	**Compound 10** **2(1H)-Pyrimidinone, 5-fluoro-3,4-dihydro-4-thioxo**	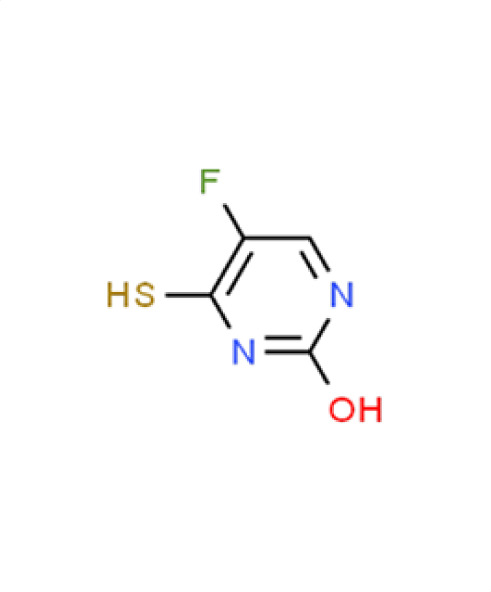
*C. albicans*	−1.1	9.5		
*C. auris*	−0.1	19.9		
***N. glabratus***	**−2.1**	**53.1**		
*C. neoformans*	−0.8	29.4		
*A. fumigatus*	−0.3	31.2	**Compound 11** N-methyl-(5-pyrid-4-ylthien-2-yl)methylamine	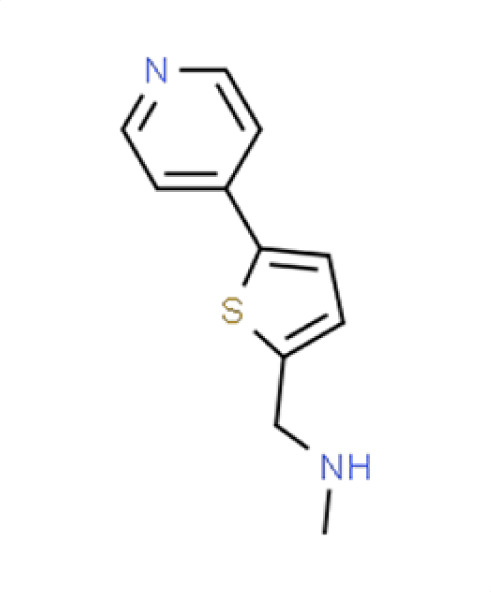
*C. albicans*	0.2	1.6		
*C. auris*	−1.4	48.5		
***N. glabratus***	**−3.7**	**86.7**		
*C. neoformans*	−0.7	21.6		
*A. fumigatus*	−0.7	35.8	**Compound 12** 4-(4-[Trifluormethyl]phenyl)−1,2,3-thiadiazol-5-amine	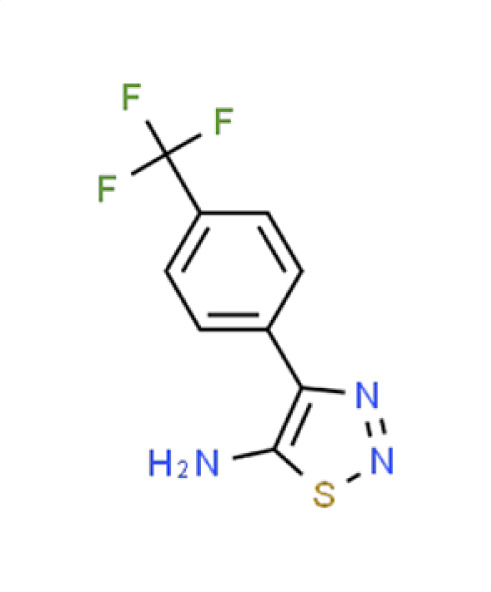
*C. albicans*	−0.6	8.1		
***C. auris***	**−2**	**66.3**		
*N. glabratus*	−1.7	54.7		
*C. neoformans*	−1.7	35.8		
*A. fumigatus*	0.7	13.2	**Compound 13** 3-amino-1-(3-fluorophenyl)thiourea	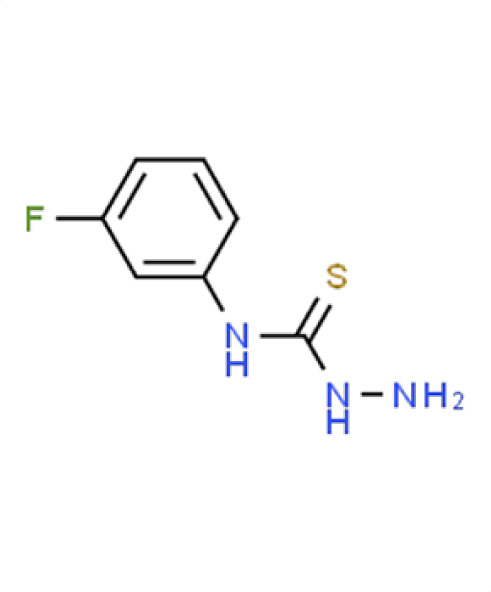
*C. albicans*	−0.1	8.2		
***C. auris***	**−2.2**	**74.3**		
*N. glabratus*	0.7	−8.5		
*C. neoformans*	−0.2	15.9		
*A. fumigatus*	−0.2	29	**Compound 14** Methyl 5-amino-1-benzothiophene-2-carboxylate	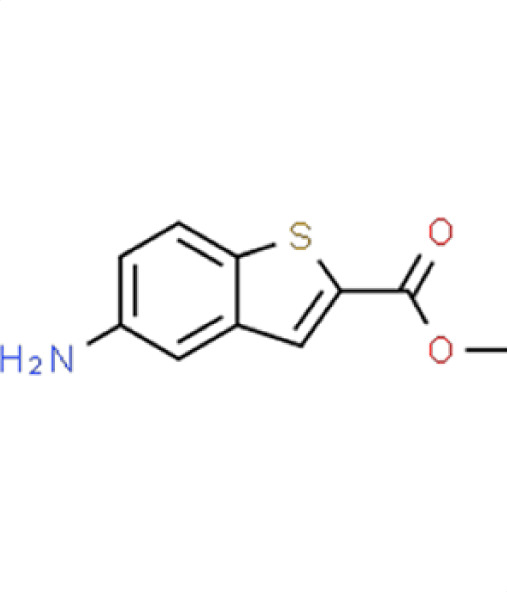
*C. albicans*	−0.1	4		
***C. auris***	**−2.2**	**73.5**		
*N. glabratus*	0	6.3		
*C. neoformans*	−0.2	14.9		
*A. fumigatus*	−0.3	27.2	**Compound 15** **2-amino-4-(3,4-difluorophenyl)thiazole**	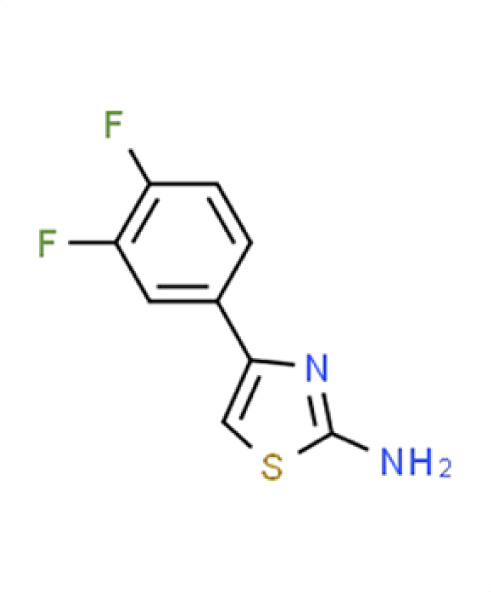
*C. albicans*	0.2	1.7		
*C. auris*	−1.2	47.2		
***N. glabratus***	**−2.2**	**61.8**		
*C. neoformans*	0.5	−1.5		
*A. fumigatus*	0.5	9.9	**Compound 16** **N-methyl-N-(3-pyridin-3-ylbenzyl)amine**	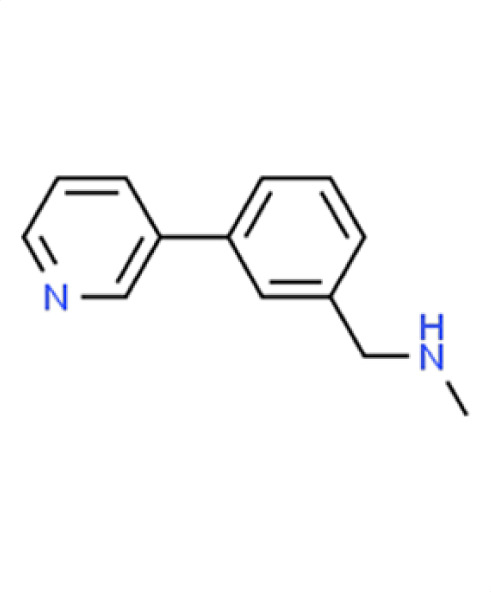
*C. albicans*	0.4	0		
*C. auris*	−1.8	61.9		
***N. glabratus***	**−2.3**	**62**		
*C. neoformans*	−0.1	11.3		
*A. fumigatus*	−1.6	53	**Compound 17** Ethyl 4-acetyl-3-methyl-5-(methylthio)thiophene-2-carboxylate	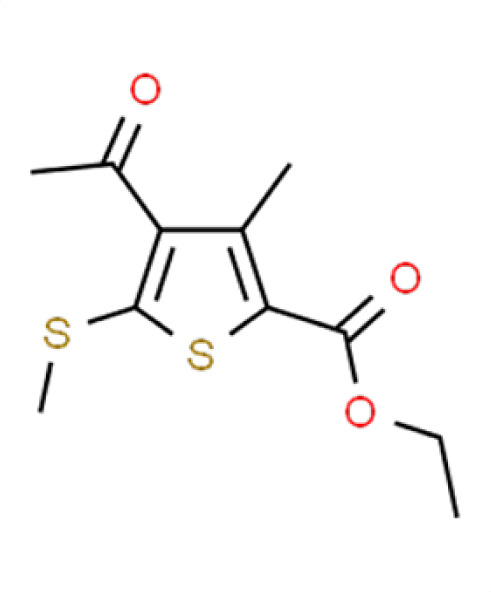
*C. albicans*	−0.2	8		
***C. auris***	**−2**	**68.4**		
*N. glabratus*	−1.4	43.4		
*C. neoformans*	−0.5	19.2		
*A. fumigatus*	2.6	−5.5	**Compound 18** 4,4′-Thiobisphenol	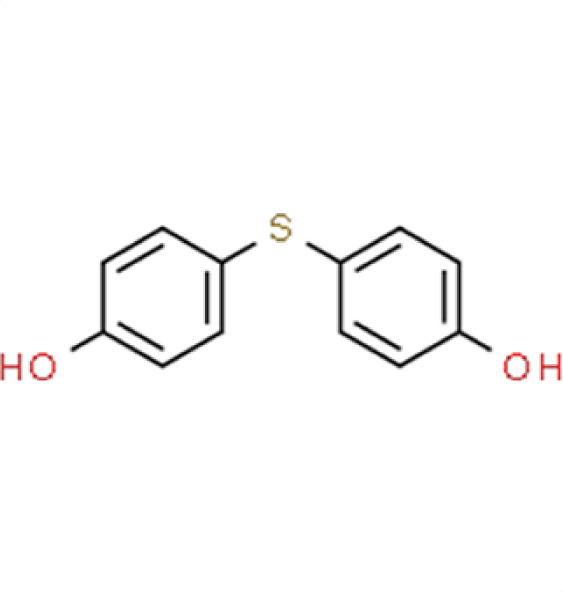
*C. albicans*	−1.1	9.5		
*C. auris*	−0.08	19.5		
***N. glabratus***	**−2.4**	**53.1**		
*C. neoformans*	−0.8	29.4		

^*a*^ Text highlighted in bold indicates best species hit as determined by *z*-score.

PAINs include isothiazolone chemical groups (Table S3) and resulted in a complete inhibitory effect across all species, making them easy to distinguish from the list of potentially useful compounds. Other toxic compounds such as dazomet and dimethachlon are more variable in their inhibitory activity but are both well-known soil fumigants and pesticides and are not suitable for medicinal use. 1-Nitroso-2-naphthol was indicated as a possible toxic compound due to its pan-fungal inhibitory activity, which was confirmed by a medicinal chemist (personal communication) to have DNA mutagenic properties.

## DISCUSSION

Here, we establish a new and promising phenotypic approach for pan-fungal drug discovery that is simple, adaptable, and amenable to higher throughput screening. Through *z*-factor analysis, we have demonstrated that our assays are high quality and sensitive enough to identify potentially useful hits with a standardized *z*-score parameter. Therefore, with minimal time, resources, and effort, the early stages of drug discovery can be fast-tracked and standardized across multiple fungal species. *A. fumigatus*, *C. albicans*, *C. auris*, *C. neoformans*, and *N. glabratus* have been internationally recognized as pathogens of critical or high priority (19).

In the early stages of antifungal drug discovery, phenotypic screens often focus on single species or genera before assessing broad-spectrum potential with a small subset of compound hits. This approach has been applied successfully in the case of olorofim (F901318), which was discovered through *in vitro* screening of a large library of small molecules against *A. fumigatus*. The initial hits were pooled and tested on *Candida* species, where the compounds had no antifungal activity (30). Notwithstanding the success of the olorofim discovery campaign, it remains feasible that a multi-species approach may enhance the chances of identifying novel drug targets having pan-fungal relevance. In our pan-fungal approach, we identified several compounds that exhibited multi-species activity but did not meet the *z*-score threshold in all cases. Such compounds may eventually yield useful antifungals, for example, in cases where the compound target is conserved, but the binding affinity or cell penetration differs across species, which may be overlooked in a conventional single-species approach. While such insights cannot be ascertained through phenotypic screening, where the target is unknown, compounds could be identified via structural activity relationship studies combined with target identification methods (e.g., direct biochemical, genetic interaction, or computational) and then further evolved (31).

Historically, phenotypic screening was considered unsuitable for FBDD due to concerns that these types of assays would lack the sensitivity to detect hits among the inherently weak binding affinities of chemical fragments (32, 33). Arguably, this might explain the overwhelming number of hits identified when using higher concentrations of chemical fragments in *C. neoformans* (Fig. S1). However, based on the excellent *z*-factor scores for all of the 0.1 mM assays, the sensitivity is reliable enough to detect outlying compounds at lower concentrations. These compounds may have novel antifungal properties and/or MOAs against one or more of the species screened in this study.

While molecular target-based screening has dominated the pharmaceutical industry since the 1980s, the emergence of improved phenotypic methods more amenable to higher throughput screening has renewed interest in this approach (8, 32–37). Phenotypic screening can revive an otherwise stagnant area in drug discovery, expanding the limitations of the previously characterized “druggable” target space with an unbiased approach. One challenge of pan-fungal phenotypic screening is the variability in growth rates and nutritional requirements across fungal species. In our methodology, we chose to use in-house media preparation (20, 21) instead of standard susceptibility testing media RPMI. One major advantage of using fRPMI over other enriched media such as yeast peptone dextrose (YPD) is the flexibility it allows for altering its composition. Where traditional RPMI 2% G-MOPS is usually provided in a ready-made format (i.e., powder or liquid media), the in-house formulation of fRPMI means that nutrient-limiting conditions can be achieved by reducing or eliminating components such as minerals or amino acids. However, this benefit must be balanced against tailoring required growth times since accelerated growth can lead to the formation of hyphal aggregates on the liquid-surface interface, which is known to be problematic in growth-based assays for filamentous fungi (38).

Some antimicrobial screening efforts utilize fluorescent or luminescent measurements to improve the sensitivity of phenotypic screens (39–41). This may be particularly beneficial for species such as *C. neoformans* and *C. parapsilosis*, which can have relatively poor growth in the microbroth format. The fRPMI did not improve growth in *C. neoformans* but was equivalent to RPMI 2% G-MOPS and still produced an excellent *z*-score. A clear limitation of optical density growth-based assays is that they are not suitable for assessing the activity of compounds on morphologies such as active hyphal growth and biofilms. Emulating these clinically relevant morphologies may arguably be more important than compound activity on ungerminated spores (42). Thus, metabolic reporters or crystal violet assays may be more appropriate in these cases. Metabolic assays usually require additional incubation times, but certain non-toxic metabolic reporters can be added to the initial inoculum (41). Our assay setup can be easily augmented to include such metabolic or viability reporters, but in the case of this pilot study, the sensitivity of the growth assay was high enough to negate the need for any additional steps. Moreover, as we have recently demonstrated, computational calibration of morphology-specific growth rates can usefully compensate for inaccuracies of indirect OD_600_ measurements (43).

In this study, except for *C. auris*, we chose laboratory strains to represent each species as they are widely used and reasonably well characterized. One caveat to this approach is that while SC5314 has been used as a reference strain for *C. albicans* in many laboratories since the mid-1980s, recently it has been shown that this strain exhibits highly filamentous and invasive characteristics, owing to a rare dominant allele of regulator of biofilm 1 (*ROB1*) (44). Furthermore, while there are laboratory reference strains for *A. fumigatus*, *C. albicans*, *N. glabratus*, and *C. neoformans*, there does not seem to be a representative strain when it comes to *C. auris*. The strain that we selected (470036) is a fluconazole-resistant clinical isolate from clade I. Clade-specific differences in metabolics, stress responses, and susceptibility mean that this single isolate may not represent the behavior of other *C. auris* clades or isolates that have aggregating phenotypes (45, 46).

Our study was heavily biased toward yeast species, with only one filamentous mold chosen for full library screening. The inclusion of *Rhizopus* or other species of *Mucor* would have been a valuable inclusion in the pan-fungal screen: first, to represent non-*Aspergillus* molds and secondly to find potentially useful hits in a genus that is severely limited in treatment options. Even among the newly developed first-in-class antifungals fosmanogepix, ibrexafungerp, and olorofim, the activity of these compounds against *Rhizopus* and *Mucor* is variable at best (47).

In our proof-of-principle study, we have demonstrated a simple and reproducible pan-fungal screening platform using an enriched and adaptable media to support fungal growth in high throughput. Future work would involve the inclusion of more fungal species, particularly those that are rarer or underrepresented in drug discovery. This would enable the adoption of standardized screening strategies across a broader sample of pathogens and isolates and expand the repertoire of species that are amenable to assay. Accordingly, the simplicity and flexibility of our pilot screens open the doors to a plethora of future studies: including combinations with approved antifungals for chemo-sensitization or synergy, combining or linking chemical fragments, and studying other host-relevant or nutrient-limiting conditions.

## MATERIALS AND METHODS

### Fungal isolates and culture

A mix of clinical, environmental, and laboratory strains were used as representative fungal pathogens (Table 1). All fungal isolates were maintained at −80°C in 25% glycerol broth before use. For mold species (*Aspergillus*, *Rhizopus*, and *Lichtheimia*), aliquots of the frozen stocks were cultured on Sabouraud dextrose agar at 37°C for 48–72 hours. Conidia were harvested using sterile double deionized H_2_O (ddH_2_O) supplemented with 0.05% Tween 20, filtered through sterile Miracloth (Merck), diluted 1:200, and counted using a hemocytometer. Yeast species (*Cryptococcus*, *Candida*, and *Nakaseomyces*) were cultured on YPD agar at 30°C for 48 hours. Five individual colonies were picked and inoculated into YPD broth for an overnight culture at 30°C, shaking at 200 rpm. Yeast suspensions were centrifuged for 5 minutes at 3,000 rpm, the supernatant was discarded, and the yeast pellet was resuspended in sterile ddH_2_O. These suspensions were diluted to 1:200 in sterile ddH_2_O and counted using a hemocytometer.

### Media conditions and assay optimization

Fungal RPMI (20, 21) incorporates elements of RPMI-1640, Aspergillus Minimal Media, Dulbecco’s Modified Eagle Medium, and unique components for enhancing fungal growth. A detailed list of components with final concentrations and stock solution preparations is provided in Table 3. One minor change from the original recipe was substituting the iron source from iron phosphate (FePO_4_) to iron (III) chloride (FeCl_3_) to improve solubility. To assess the growth capabilities of fRPMI, 12 species of fungal pathogens were selected to represent both molds and yeasts (Table 1), comparing growth to EUCAST susceptibility testing media RPMI (15, 16). Briefly, the RPMI-1640 was supplemented with glucose to 2% and buffered at pH 7 using MOPS at a final concentration of 0.165  mol/L (RPMI 2% G-MOPS). One hundred microliter double strength fRPMI or RPMI 2% G-MOPS was aliquoted into the wells of each 96-well plate with either 100 µL of fungal inoculum (double concentration of 5 × 10^5^ cfu/mL) or 100 µL sterile ddH_2_O as a background control.

**TABLE 3:** Individual component concentrations for fRPMI (20, 21)^*a*^

Component	Final concentration in fRPMI (mM)	Stock solution (mg/L) (× final concentration)
Amino acids		
Glycine	0.133	1,000 (100×)
L-arginine	1.149	20,000 (100×)
L-asparagine	0.379	5,000 (100×)
L-aspartic acid	0.150	2,000 (100×)
L-cystine dihydrochloride	0.208	65,000 (1,000×)
L-glutamic acid	0.136	2,000 (100×)
L-glutamine	2.055	15,000 (50×)
L-histidine	0.097	1,500 (100×)
L-hydroxyproline	0.153	2,000 (100×)
L-isoleucine	0.382	5,000 (100×)
L-leucine	0.382	5,000 (100×)
L-lysine hydrochloride	0.219	4,000 (100×)
L-methionine	0.101	1,500 (100×)
L-phenylalanine	0.091	1,500 (100×)
L-proline	0.174	2,000 (100×)
L-serine	0.286	3,000 (100×)
L-threonine	0.168	2,000 (100×)
L-tryptophan	0.025	500 (100×)
L-tyrosine disodium salt dehydrate	0.111	2,900 (100×)
L-valine	0.171	2,000 (100×)
Vitamins^*b*^		
Biotin	0.0008	20 (100×)
Choline chloride	0.0214	300 (100×)
D-calcium pantothenate	0.0005	25 (100×)
Folic acid	0.0023	100 (100×)
Niacinamide	0.0082	100 (100×)
Para-aminobenzoic acid	0.0073	100 (100×)
Pyridoxine hydrochloride	0.0049	100 (100×)
Riboflavin	0.0005	20 (100×)
Thiamine hydrochloride	0.0030	100 (100×)
Vitamin B12	0.000004	0.50 (100×)
i-Inositol	0.1944	3,500 (100×)
Inorganic salts		
Ammonium tartrate^*b*^	5.00	92,074 (100×)
Magnesium Sulfate (MgSO_4_) anhydrous	0.41	4,884 (100×)
Sodium chloride (NaCl)^*b*^	103.45	60,000 (10×)
Sodium bicarbonate (NaHCO_3_)	23.81	20,000 (10×)
Potassium chloride (KCl)	5.33	4,000 (10×)
Sodium phosphate dibasic (Na_2_HPO_4_) anhydrous	5.63	8,000 (10×)
Trace elements		
Sodium borate (Na_2_B_4_O_7_.10H_2_O)	0.04	40 (1,000×)
Copper sulfate (CuSO_4_.5H_2_O)	0.001602	4,000 (1,000×)
Iron chloride (FeCl_3_)	0.0042781	700 (1,000×)
Manganese sulfate (MnSO_4_.7H_2_O)^*b*^	0.80	800 (1,000×)
Sodium molybdate (Na_2_MoO_4_. 2H_2_O)	0.80	800 (1,000×)
Zinc sulfate (ZnSO_4_. 7H_2_O)^*b*^	8.00	8,000 (1,000×)
Other components		
D-glucose^*b*^	111.1	200,000 (10×)
Glutathione	0.0033	1,000 (1,000×)
3-(N-morpholino)propanesulfonic acid (MOPS, pH 7)	165	345,300 (10×)
Calcium chloride (CaCl_2_)^*b*^	0.42	4,660 (100×)

^*a*^ Amino acids, vitamins, and some inorganic salts can be prepared as mixed solutions. Trace elements and other components can be prepared as individual stock solutions. Components highlighted in gray should be prepared separately from the main stocks.

^*b*^ Solutions that can be autoclaved, all other components should be filter sterilized using a 0.2 µM filter.

Kinetic growth curves for the 12 fungal species were carried out in both media using a Tecan Spark microplate reader at 37°C in ambient air. Mold species were incubated without agitation, whereas yeast species were shaken at 200 rpm for 30 seconds before measurements were taken. Data were collected in 3–4 biological replicates as OD_600_ (mold) or OD_530_ (yeast) readings every 30 minutes for 48 hours. End point measurements at 24 and 48 hours were recorded for each species using the data obtained from the kinetic growth curves.

### Antifungal susceptibility testing

MICs for either voriconazole (Fisher Scientific) or amphotericin B (EDQM), selected according to their therapeutic relevance and fungicidal activity, were used as positive controls for fungal growth inhibition. MIC testing was only carried out on the five fungal pathogens selected for screening (*A. fumigatus*, *C. albicans*, *C. auris*, *C. neoformans*, and *N. glabratus*). MICs were determined using the broth microdilution method according to EUCAST (15, 16) guidelines, using fRPMI as well as RPMI 2% G-MOPS in the same manner. The concentration ranges tested were 0.03–16 µg/mL for both voriconazole and amphotericin B as a twofold dilution series. MICs were recorded by visual inspection for *A. fumigatus* or OD_530_ for yeast species as a 90% or 80% reduction in growth (MIC_90_/MIC_80_) compared to the untreated control. While voriconazole is typically recorded as an MIC_50_ in yeast species, we elected for an MIC_80_ as an acceptable threshold for growth inhibition. All experiments were performed in biological triplicate and repeated on two independent days.

### Maybridge Ro3 fragment library

A selection of 500 compounds was purchased from the Maybridge Ro3 Fragment Library (Thermo Fisher), representing the chemical diversity of the larger library (2,500 compounds). Compounds were pre-dissolved in 100% DMSO to 200 mM and stored in racked glass vials. Intermediary stocks of 100 mM and 10 mM were diluted using 100% DMSO into separate 96-well microplates. All microplates used were Corning brand (96 wells, clear, flat-bottomed, and TC-treated). All stocks and aliquots of compounds were stored at −80°C until ready for further use.

### Compound library screen

For the full library screen, 2 µL or a 1:100 dilution of the 10 mM or 100 mM intermediary compound stocks were aliquoted into separate 96-well microplates. This gave a final compound concentration of 0.1 and 1 mM, respectively, with a DMSO concentration of 1%. The first and last columns of the microplates were used for positive and negative controls. For the negative control, 2 µL of DMSO was added to the first column of each microplate to a final concentration of 1%. The fungal inoculum was diluted into fRPMI to a final concentration of 2.5 × 10^5^ cfu/mL, and 198 µL was aliquoted immediately into the compound and negative control columns. For the positive control, stocks of voriconazole and amphotericin B were prepared at MIC value for each species according to the EUCAST protocol (15, 16) but then diluted 1:100 into 1× fRPMI. The fungal inoculum was diluted into the media as described above and aliquoted immediately in the final column. All 96-well plates were incubated at 37°C in ambient air without agitation. For each species, OD_600_ or OD_530_ readings were taken at optimal time points as determined by the kinetic growth curves. OD data from ±2 hours was recorded to account for any growth variation, except *C. neoformans*, which was recorded at 24 and 48 hours.

### Data analysis

*Z*-factors are a statistical measure to assess the quality of an assay, based on the separation between positive (no growth) and negative (untreated growth) controls and the SDs between them. An assay that scores ≥0.5–1 is considered excellent, meaning that it is sensitive enough to distinguish hits reliably. *Z*-factors were calculated on a plate-by-plate basis for each species for the full library screen, providing an overall mean and a range for plate-to-plate variability. Each species screen consisted of 21–27 96-well plates, which include 6–8 positive and 6–8 negative controls per plate and three biological replicates.


$$Z\;factor\;=\;\;\frac{3\sigma_{C+}\left(3SD\;positive\;signal\right)-3\sigma_{C-}\left(3SD\;negative\;signal\right)}{3\mu_{C+}\left(positive\;mean\right)\;–3\mu_{C-+}\left(negative\;mean\right)}.$$

A standard score, or “*z*-score,” is a statistical measure that indicates the number of SDs a data point is above or below the data set mean. Assuming normal distribution, *z*-scores of ≤−1.96 and ≤−2.58 indicate growth reduction with 95% and 99% confidence, respectively. Growth inhibition was also recorded as a percentage compared to the untreated control.


$$Z\text{-}score\;=\;\;\frac{x\;\left(sample\;value\right)-\mu \;\left(mean\;of\;all\;samples\right)}{\sigma \;\left(standard\;deviation\;of\;all\;samples\right)}.$$

## SUPPLEMENTAL MATERIAL

Supplemental figures aac.01328-24-s0001.docx Figures S1 and S2.

Supplemental tables aac.01328-24-s0002.docx Tables S1 to S3.

ASM does not own the copyrights to Supplemental Material that may be linked to, or accessed through, an article. The authors have granted ASM a non-exclusive, world-wide license to publish the Supplemental Material files. Please contact the corresponding author directly for reuse.
