# Supplementary material for: A novel pan-fungal screening platform for antifungal drug discovery: proof of principle study
Source: Antimicrob Agents Chemother. 2025 Apr 1;69(5):e01328-24. doi: 10.1128/aac.01328-24 (PMC12057344; doi:10.1128/aac.01328-24)
Supplement: Supplemental figures — Figures S1 and S2. [file aac.01328-24-s0001.docx]

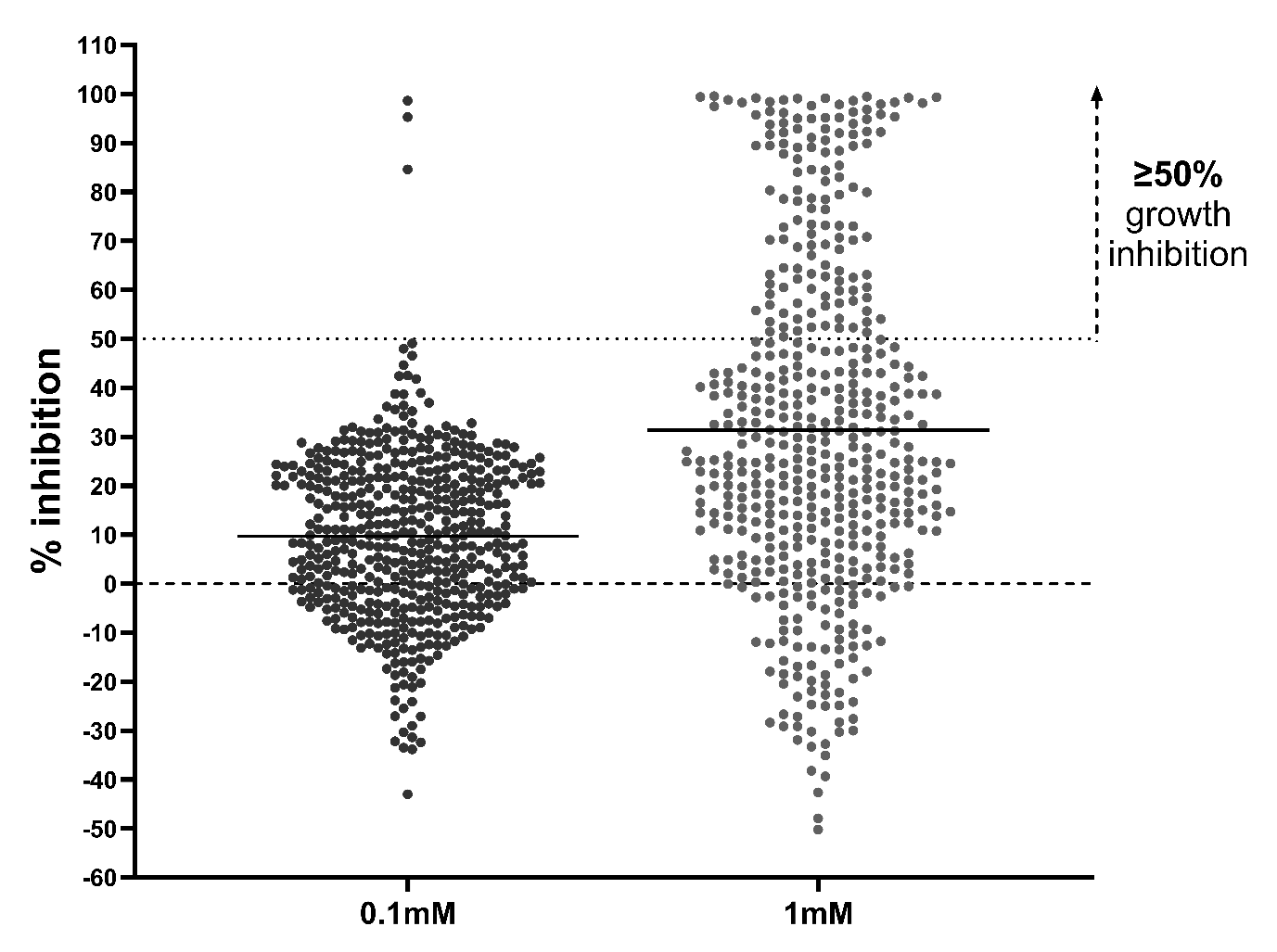
**Supplementary figure 1.** Percent inhibition plots for *Cryptococcus neoformans* H99E comparing screens from 0.1 and 1 mM chemical compound concentrations. Data points represent the mean value from 3 biological replicates. Dotted lines indicate 0% and 50% inhibition compared to an untreated control. Solid lines indicate dataset mean.

**Supplementary figure 2.** Percent inhibition plots for full chemical fragment library screens. Data points represent the mean value from 3 biological replicates. Dotted lines are indicated at 0% and 50% inhibition compared to the untreated control
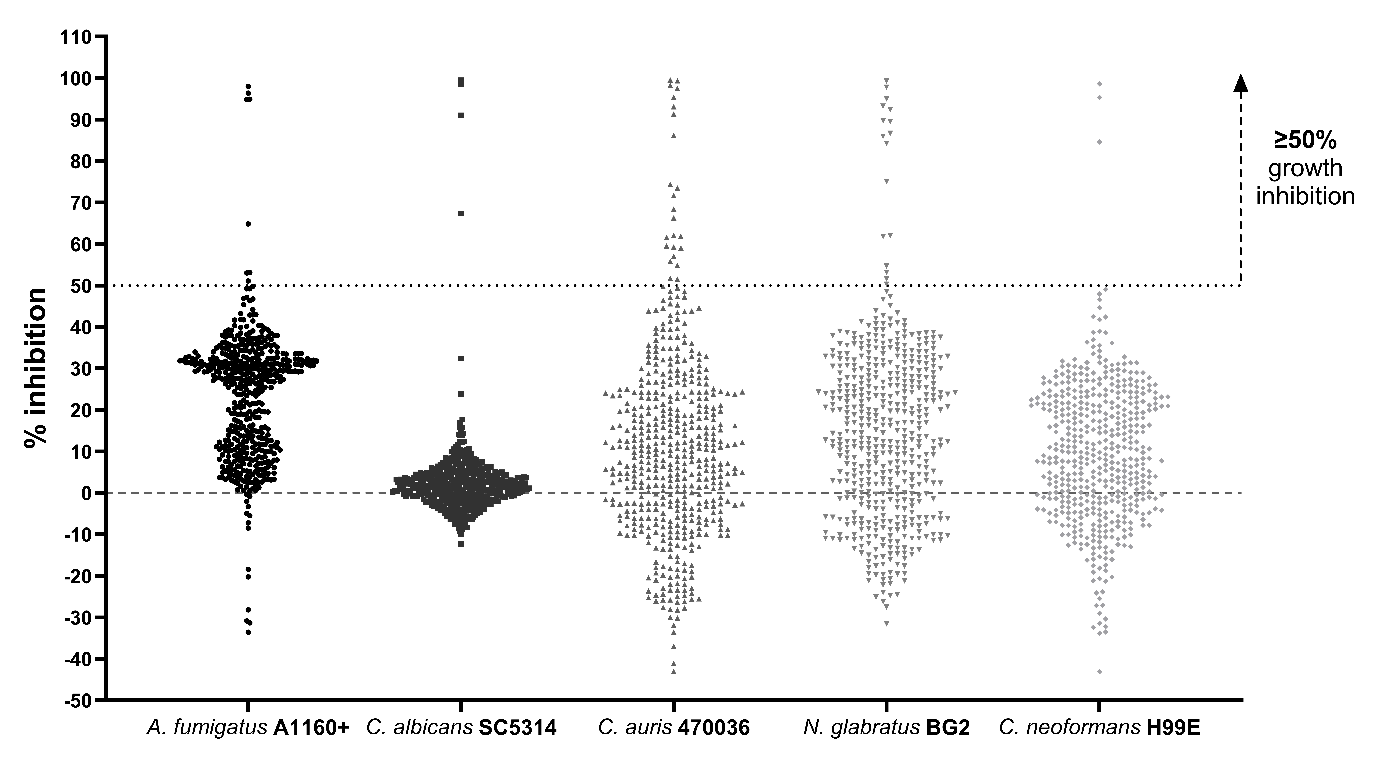
.
