# Supplementary material for: A novel pan-fungal screening platform for antifungal drug discovery: proof of principle study
Source: Antimicrob Agents Chemother. 2025 Apr 1;69(5):e01328-24. doi: 10.1128/aac.01328-24 (PMC12057344; doi:10.1128/aac.01328-24)
Supplement: Supplemental tables — Tables S1 to S3. [file aac.01328-24-s0002.docx]

# **Supplementary tables**

**Table S1.** Minimum inhibitory concentrations (MICs) for five organisms used in chemical fragment screening in fungal RPMI (fRPMI) and RPMI 2% G-MOPS. Data indicates MIC_90_ values recorded at 24 and 48 hours, except those marked with an asterisk* which are read as MIC_80_.

| **Organism/strain** | **Amphotericin B MIC_90_ (µg/ml)** | | | | **Voriconazole MIC_90_/MIC_80_ (µg/ml)** | | | |
| --- | --- | --- | --- | --- | --- | --- | --- | --- |
|  | **RPMI 2% G-MOPS** | | **fRPMI** | | **RPMI 2% G-MOPS** | | **fRPMI** | |
|  | 24 hrs | 48hrs | 24 hrs | 48hrs | 24 hrs | 48hrs | 24 hrs | 48hrs |
| *Aspergillus fumigatus* A1160^+^ | 0.5 | 1 | 1 | 2 | 0.125 | 0.25 | 0.125 | 0.25 |
| *Candida albicans* SC5314 | 0.5 | 1 | 1 | 2 | 0.03* | 0.03* | 0.03* | 0.03-0.06* |
| *Candida auris* 470036 | 0.5 | 1 | 1 | 2 | 1* | 2* | 2* | 4* |
| *Nakaseomyces glabratus* (*Candida glabrata*) BG2 | 0.5 | 1 | 1 | 2 | 0.25* | 0.5* | 0.25* | 0.5* |
| *Cryptococcus neoformans* H99E | 0.25 | 0.5 | 0.5 | 1 | 0.25* | 0.25* | 0.25* | 0.5* |

| **Plate number** | ***A. fumigatus* A1160^+^** | ***C. albicans* SC5314** | ***C. auris* 470036** | ***N. glabratus* BG2** | ***C. neoformans* H99E** |
| --- | --- | --- | --- | --- | --- |
| 1 | 0.585307963 | 0.843081 | 0.852632 | 0.847479 | 0.608036136 |
| 2 | 0.565224613 | 0.851089 | 0.837413 | 0.863784 | 0.626907541 |
| 3 | 0.600394897 | 0.839201 | 0.821519 | 0.868672 | 0.612221917 |
| 4 | 0.619314141 | 0.838734 | 0.843892 | 0.881295 | 0.662004375 |
| 5 | 0.586657876 | 0.849912 | 0.855725 | 0.857186 | 0.638211992 |
| 6 | 0.552919373 | 0.838512 | 0.865002 | 0.844737 | 0.632609276 |
| 7 | 0.582499121 | 0.84677 | 0.866418 | 0.871431 | 0.642807791 |
| 8 | 0.562980986 | - | - | - | - |
| 9 | 0.563249558 | - | - | - | - |
| **Overall mean** | 0.579838725 | 0.8439 | 0.848943 | 0.862083 | 0.631828433 |

**Table S2.** Mean Z-factors based on plate-to-plate or day-to-day variations. Plates were split and numbered to accommodate the 500 compounds in their allocated order. Z-score values for individual plates/species represent the mean from 3 biological replicates performed on a single day. Each plate number also indicates a separate day when the experiments were performed. An overall plate mean indicates the average Z-factor for the individual organism across plates and replicates.

**Table S3.** Pan assay interference and toxic compounds removed from the list of compound hits.

| **Organism** | **Z-score** | **% inhibition vs. untreated control** | **Chemical name** | **Chemical properties** | **Chemical structure** |
| --- | --- | --- | --- | --- | --- |
| *A. fumigatus* | -3.9 | 98 | **PAIN compound 1**  2-(3-chloro-4-fluorophenyl)-1,2-thiazol-3(2*H*)-one | Pan-assay interference compound, broad spectrum biocide | 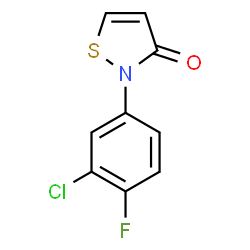 |
| *C. albicans* | -10.5 | 98.5 |  |  |  |
| *C. auris* | -3.1 | 98.2 |  |  |  |
| *N. glabratus* | -4.3 | 97.8 |  |  |  |
| *C. neoformans* | -4.5 | 95.3 |  |  |  |
|  | | | | | |
| *A. fumigatus* | -3.8 | 96.4 | **PAIN compound 2**  2-[3-(Trifluoromethyl)phenyl]-1,2-thiazol-3(2H)-one | Pan-assay interference compound, broad spectrum biocide | 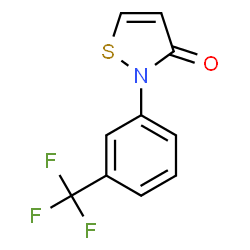 |
| *C. albicans* | -7.4 | 99.5 |  |  |  |
| *C. auris* | -3.1 | 99.6 |  |  |  |
| *N. glabratus* | -4.4 | 99.3 |  |  |  |
| *C. neoformans* | -4.7 | 98.6 |  |  |  |
|  | | | | | |
| *A. fumigatus* | -3.8 | 94.9 | **TOXIC compound 1**  Dazomet | Soil fumigant, broad toxicity | 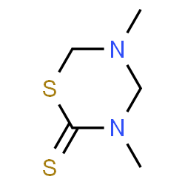 |
| *C. albicans* | -7.1 | 67.4 |  |  |  |
| *C. auris* | -3.1 | 99.3 |  |  |  |
| *N. glabratus* | 1.6 | -12.9 |  |  |  |
| *C. neoformans* | 1.1 | -10 |  |  |  |
|  | | | | | |
| *A. fumigatus* | -1.4 | 49.2 | **TOXIC compound 2**  Dimethachlon | Pesiticide, environmental fungicide | 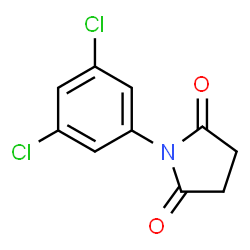 |
| *C. albicans* | 0 | 5 |  |  |  |
| *C. auris* | -2.9 | 93.1 |  |  |  |
| *N. glabratus* | 0.7 | 4.3 |  |  |  |
| *C. neoformans* | -2.1 | 49.1 |  |  |  |
|  | | | | | |
| *A. fumigatus* | -3.8 | 94.8 | **TOXIC compound 3**  1-Nitroso-2-naphthol | Metal chelator, electrophillic DNA mutagen | 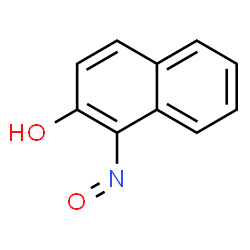 |
| *C. albicans* | -9.7 | 91 |  |  |  |
| *C. auris* | -3 | 95.4 |  |  |  |
| *N. glabratus* | -3.6 | 86 |  |  |  |
| *C. neoformans* | -4 | 84.6 |  |  |  |
